# The Role of Obesity in the Poor Prognosis of COVID-19 Infection: A Review of 192 Patients

**DOI:** 10.1155/2024/7212355

**Published:** 2024-05-13

**Authors:** Ziad Feghaly, Rany Aoun, Christian Mouawad, Bilal Chamaa, Houssam Dahboul, Serge Kassar, Michael Osseis, Roger Noun, Ghassan Chakhtoura

**Affiliations:** Department of Digestive Surgery, Hotel Dieu de France Hospital, Saint Joseph University, Beirut, Lebanon

## Abstract

**Methods:**

We performed a retrospective study on all patients having COVID-19 infection and admitted to our institution between March 2020 and June 2021. Inclusion criteria included any patient over the age of 18 admitted to our institution's COVID-19 unit, or intensive care unit, with a positive COVID-19 PCR or positive COVID-19 serology (IgM).

**Results:**

192 patients met the inclusion criteria, with an average age of 62.68 years and a slight male predominance (64.58%). 76.04% of hospitalized patients and 80% of those admitted to the ICU were either overweight or obese. No statistically significant difference was found regarding the risk of in-hospital mortality and invasive ventilation. The same applies to the length of stay, admission to intensive care, O_2_ needs, and for the various complications (all *p* values were >0.05). Patients with obesity type II and III have an increased risk of cardiac arrests and need for intubation and mechanical ventilation.

**Conclusion:**

Obesity tends to be a major risk factor for a pejorative evolution in the COVID-19 infection.

## 1. Introduction

In December 2019, the city of Wuhan in China was the starting point of an epidemic of pneumonia. Subsequently, Chinese researchers detected a new virus from the coronavirus family, SARS-CoV-2 (severe acute respiratory coronavirus 2) in affected individuals [1, 2]. Then, on January 30, 2020, the World Health Organization (WHO) considered the outbreak of COVID-19 to be an international public health emergency [3].

Since its emergency, COVID-19 has reached millions of cases worldwide and millions of deaths which has had a considerable impact on the health system and which has led to a significant number of hospitalizations especially in the intensive care units.

Elderly people with comorbidities such as chronic lung problems, cardiovascular disease, kidney failure, diabetes, and arterial hypertension were more vulnerable to a pejorative evolution of the infection with a higher mortality rate. A recent report from Centers for Disease Control and Prevention (CDC) showed that the most common conditions among people hospitalized with COVID-19 were diabetes, chronic lung problems, and cardiovascular disease [4].

Simultaneously, obesity has been a major health problem for many years and continues to worsen. WHO considers obesity as a pandemic with more than 1.9 billion adults overweight and more than 650 million obese [5].

These two pandemics seem to be converging since obesity increasingly appears to be a poor prognostic factor for COVID-19 infection. In parallel, obesity is well known to be a risk factor for cardiovascular disease, diabetes, and high blood pressure. Moreover, numerous respiratory complications have been associated with obesity, including increased demand for ventilation, increased breathing effort, and decreased respiratory compliance [6].

In addition, similar observations have been noted in other respiratory viruses such as during the influenza A (H1N1) pandemic in 2009. Obese patients with this virus had a higher risk of hospitalization and death, with a longer duration of hospitalization and mechanical ventilation compared to individuals with normal weight [7, 8].

This also seems to have been applied during the COVID-19 pandemic. A growing number of studies are trying to establish a link between obesity and the severity/mortality of SARS-CoV-2 infection. For instance, a study performed in the United States on a group of 3615 COVID-19 patients, under the age of 60, showed that patients with a body mass index (BMI) between 30 and 34, and those with a BMI > 35, were, respectively, 1.8 and 3.6 times more likely to be admitted to intensive care units (ICU) than those with a normal weight [9].

Similarly, a retrospective study from New York found that obese and overweight people had an increased risk of mortality and intubation compared to those with a BMI < 25 kg/m^2^ [10].

In this study, we aimed to evaluate the associations between the BMI and the risk of in-hospital death, the need of mechanical ventilation, length of hospital stays, oxygen needs, admission to the ICU (intensive care unit), and the different complications in patients admitted with COVID-19 at our institution.

## 2. Methods

### 2.1. Study Design

This is a retrospective study that was performed after getting the ethics committee's agreement.

### 2.2. Setting

Data analysis was performed on the records of patients admitted to the Hôtel Dieu de France hospital (HDF) in Beirut for an active COVID-19 infection between March 2020 and June 2021.

### 2.3. Participants and Variables

Inclusion criteria are (1) patients over the age of 18, (2) admitted to the COVID unit or ICU, (3) with a positive COVID-19 PCR, or COVID-19 serology (IgM) (4) and whose data are available for BMI, age, and admitting and discharging dates.

### 2.4. Data Sources/Measurement

The diagnosis of an active COVID-19 infection was defined by a polymerase chain reaction testing evidence of severe acute respiratory syndrome coronavirus 2 (SARS-CoV-2) RNA or by the presence of specific IgM antibodies of COVID-19.

The classification criteria were the BMI [calculated by the formula weight (kg)/height^2^(m^2^)], the parameters of which were collected from the patient files on the DxCare® software, and the patients for whom these data were not available were excluded from the study. Patients were then divided into 4 groups according to BMI: normal weight (BMI ≤ 25), overweight (25 < BMI < 30), obese type I (30 < BMI < 35), and obese type II-III (BMI ≥ 35). The group of individuals with normal weight was designated as the reference group for the statistical analysis.

Primary outcomes were intrahospital death and the need for mechanical ventilation. Secondary outcomes include the length of hospital stay, admission to the intensive care unit, duration of mechanical ventilation, O_2_ needs (in liters), and various complications (acute renal failure, need for dialysis, heart failure, myocardial infarction, stroke, cardiac arrest, deep vein thrombosis, pulmonary embolism, and shock).

### 2.5. Bias

The size of the sample studied, which was limited to 192 patients, is relatively not too much compared to the other studies in this field.

### 2.6. Statistical Methods

Statistical analysis was carried out using SPSS IBM Statistics for Windows version 21. Descriptive statistics were presented in percentage for categorical variables and mean for continuous variables. To test the associations between the BMI and the risk of in-hospital death, the need of mechanical ventilation, length of hospital stays, oxygen needs, admission to the ICU (intensive care unit), and the different complications, we performed the chi-square test for categorical variables and one-way ANOVA for continuous variables. We performed a logistic regression adjusting for age, sex, and tobacco use for the outcomes that were statically significant. A significant difference is considered when the *p* value is <0.05.

## 3. Results

### 3.1. Population Characteristics

362 patients were screened, and 192 were included in this study. The rest of the patients were those whose hospital stay was short and were not admitted to the intensive care unit.  Table 1 summarizes the main characteristics of the population studied, divided into three groups according to the BMI.

We note that 76.04% of patients hospitalized at our institution for COVID-19 were either overweight or obese, compared to only 23.96% who had a normal weight. The average age for all groups combined was 62.68 years with a male predominance (64.58%) and a high prevalence of comorbidities such as diabetes, hypertension, and dyslipidemia.

### 3.2. Outcomes

32 in-hospital deaths were registered. Half of these deaths occurred in obese patients, whereas only 8 deaths in patients with BMI < 25. This difference in death among weight categories was not statistically significant (*p* value = 0.127). As for mechanical ventilation, a statistically significant difference is noted (38.1% in obese type II-III, 14.8% in obese type I, 26.8% in overweight individuals, and 10.9% for normal weight patients) (*p* value = 0.026) (Table 2).

We did not find any statistically significant difference concerning the length of stay among the four groups (*p* value = 0.632) with an average of 16.6% in obese type II-III, 15.7% in obese type I, 21% in overweight individuals, and 17.2% for normal weight patients (Table 2).

We noted that 41.67% of COVID-19 patients were admitted to the intensive care unit with no significant difference among weight categories (Table 2). According to oxygen needs, no difference was noted.

After adjustment for age, sex, and tobacco use, overweight participants and participants with a BMI ≥ 35 kg/m^2^ were more likely to need intubation compared to participants with a normal BMI, OR 3.11 (1.01, 9.58), and OR 5.69 (1.44, 22.51), respectively (Table 3).

### 3.3. Complications

No statistically significant difference was registered between weight categories (*p* value = 0.508) according to complications. However, compared to the other groups, obese patients with a BMI > 35 had more need for intubation (*p* value 0.013) and more cardiac arrests, even if this last one was not statistically significant (*p* value >0.05).

Likewise, for all the other complications cited in Table 3, no significant difference was found (all the other *p* values were greater than 0.05).

## 4. Discussion

Reviewing medical literature, a descriptive study of a group of 24 patients from Seattle, Washington (13 of those were obese), showed that 85% of the obese people needed intubation and 62% died. These percentages are higher than those of nonobese patients, where 64% required mechanical ventilation and 36% died [11].

Subsequently, several studies were carried out on the same subject but with a larger number of patients. A study conducted in New York on 5279 individuals showed that a patient with BMI > 40 has a greater risk of hospitalization and severe infection [12].

Another study published in the journal of the AHA (American Heart Association), including 7606 patients, showed that obese patients are more likely to be hospitalized with COVID-19 and have a higher risk of in-hospital death and mechanical ventilation, especially if they are young (age ≤50 years). Obese patients are also at a greater risk of venous thromboembolism and dialysis [13].

Additionally, in a CDC (Center of disease control) report, among 148,494 adults who were diagnosed with COVID-19 in 238 US hospitals between March and December 2021, 71,491 were hospitalized. Of those admitted, 78% were either overweight or obese [14]; this result is comparable to that observed in our study where 76% of hospitalized patients belonged to these two categories. This percentage is significantly higher than that observed in the Lebanese population in general where it is estimated that 53% of Lebanese have a BMI > 25 [15]. So, in Lebanon, obesity could be a risk factor for the need for hospitalization in the event of COVID-19, but there is no statistical evidence to confirm this hypothesis.

Several meta-analyses have also supported the hypothesis that obesity is considered a risk factor for hospitalized patients. For instance, an analysis of 24 retrospective studies revealed that obesity is a significant risk factor for hospital admission. Intensive care (OR = 1.21, CI: 1.002–1.46) and invasive mechanical ventilation (OR = 2.05, CI: 1.16–3.64) [16].

Currently, the CDC, based on numerous studies including meta-analyses, considers obesity (BMI > 30) to be one of the comorbidities associated with a higher risk of developing severe COVID-19 infection.

Therefore, our results showed that patients with obesity type II and III have a poor prognosis in the evolution of COVID-19 infection. Compared to other patients, they risk more need for intubation and mechanical ventilation and more cardiac arrests.

## 5. Limitations of the Study

The size of the sample studied, which was limited to 192 patients, is relatively not too much compared to the other studies in this field.

## 6. Conclusion

Patients with obesity type II and III have a poor prognosis in the evolution of COVID-19 infection with an increased risk of cardiac arrests and need for intubation and mechanical ventilation. Further studies with a larger sample size are required to confirm obesity's effect on other complications.

## Figures and Tables

**Table 1:** Population characteristics, based on BMI class (*n* = 192).

Patient characteristics	BMI < 25 (*n* = 46) 24%	25 ≤ BMI < 30 (*n* = 71) 37%	30 ≤ BMI < 35 (*n* = 54) 28.1%	BMI ≥ 35 (*n* = 21) 10.9%	Total (*n* = 192)
Age (years)	60.15 [18.74]	64.56 [13.02]	62.72 [13.95]	61.81 [17.48]	62.68 [19–96]
Male sex	24 (52%)	46 (64.8%)	41 (75.9%)	13 (61.9%)	124 (64.58%)
Weight (kg)	63.8 [1.62]	79.86 [11]	90.76 [11.23]	108.3 [19.21]	82.19 [37–157]
Height (m)	1.69 [0.09]	1.70 [0.11]	1.69 [0.10]	1.67 [0.11]	1.69 [0.10]
Smoking	12 (26.1%)	16 (22.5%)	14 (25.9%)	6 (28.6%)	50 (26.04%)
Diabetes	10 (21.7%)	23 (32.39%)	16 (29.6%)	7 (33.3%)	56 (29.17%)
Hypertension	23 (50%)	43 (60.6%)	34 (63%)	14 (66.7%)	114 (59.38%)
Dyslipidemia	18 (39.1%)	32 (45.1%)	21 (38.9%)	8 (38.1%)	79 (41.1%)

**Table 2:** Comparisons of the various parameters and complications between the 3 groups studied (*n* = 192).

Outcomes	BMI < 25	25 ≤ BMI < 30	30 ≤ BMI < 35	BMI ≥ 35	Total	*P* value
Length of stay (days)	17.2 [35.1]	21.0 [23.1]	15.7 [10.35]	16.6 [20.2]	18.1 [23.7]	0.632
Admission to the ICU	16 (34.8%)	33 (46.5%)	23 (42.6%)	8 (38.1%)	80 (41.67%)	0.455
Mechanical ventilation	5 (10.9%)	19 (26.8%)	8 (14.8%)	8 (38.1%)	40 (20.8%)	0.026
Oxygen needs (>3 liters) (*n* = 145)	33.6 [29.7]	40.3 [25.4]	37.3 [27.1]	44.1 [27.8]	38.2 [27.8]	0.583
Hospital death	8 (17.4%)	8 (11.3%)	9 (16.7%)	7 (33.3%)	32 (16.7%)	0.127
Complications	12 (26.1%)	21 (29.6%)	19 (35.2%)	9 (42.9%)	61 (31.8%)	0.508
Acute renal failure	10 (21.7%)	11 (15.5%)	6 (11.1%)	6 (28.6%)	33 (17.2%)	0.248
Dialysis	2 (4.3%)	1 (1.4%)	1 (1.9%)	2 (9.5%)	6 (3.1%)	0.256
Heart failure	4 (8.7%)	1 (1.4%)	3 (3.7%)	1 (4.8%)	8 (4.2%)	0.288
Myocardial infarction	1 (2.2%)	0	2 (3.7%)	0	3 (1.6%)	0.364
Stroke	0	1 (1.4%)	1 (1.9%)	0	2 (1%)	0.767
Cardiac arrest	8 (17.4%)	9 (12.7%)	8 (14.8%)	7 (33.3%)	32 (16.7%)	0.160
Deep vein thrombosis	0	2 (2.8%)	0	1 (4.8%)	3 (1.6%)	0.294
Pulmonary embolism	1 (2.2%)	5 (7%)	2 (3.7%)	2 (9.5%)	10 (5.2%)	0.497
Shock	8 (17.4%)	10 (14.1%)	6 (11.1%)	5 (23.8%)	29 (15.10)	0.540

**Table 3:** Need for intubation in the different groups after adjustment for age, sex, and tobacco use.

	Need for intubation			
	OR	95% CI		*P* value
BMI				
Normal	Ref	Ref	Ref	
Overweight	3.11	1.01	9.58	0.048
Obese [30–35]	1.29	0.36	4.53	0.691
Obese ≥35	5.69	1.44	22.51	0.013
Age	1.04	1.01	1.07	0.009
Sex	0.63	0.27	1.45	0.279
Tobacco use	2.86	1.29	6.32	0.010

## Data Availability

The data used to support the findings of this study are available from the corresponding author upon request.
